# Gαi3-Dependent Inhibition of JNK Activity on Intracellular Membranes

**DOI:** 10.3389/fbioe.2015.00128

**Published:** 2015-09-01

**Authors:** Guillaume Bastin, Jin Ye Yang, Scott P. Heximer

**Affiliations:** ^1^Department of Physiology, Heart and Stroke, Richard Lewar Centre of Excellence in Cardiovascular Research, University of Toronto, Toronto, ON, Canada

**Keywords:** Galphai3, JNK mitogen-activated protein kinases, AGS3, RGS proteins, intracellular membranes

## Abstract

Heterotrimeric G-protein signaling has been shown to modulate a wide variety of intracellular signaling pathways, including the mitogen-activated protein kinase (MAPK) family. The activity of one MAPK family class, c-Jun N-terminal kinases (JNKs), has been traditionally linked to the activation of G-protein coupled receptors (GPCRs) at the plasma membrane. Using a unique set of G-protein signaling tools developed in our laboratory, we show that subcellular domain-specific JNK activity is inhibited by the activation of Gαi3, the Gαi isoform found predominantly within intracellular membranes, such as the endoplasmic reticulum (ER)–Golgi interface, and their associated vesicle pools. Regulators of intracellular Gαi3, including activator of G-protein signaling 3 (AGS3) and the regulator of G-protein signaling protein 4 (RGS4), have a marked impact on the regulation of JNK activity. Together, these data support the existence of unique intracellular signaling complexes that control JNK activity deep within the cell. This work highlights some of the cellular pathways that are regulated by these intracellular complexes and identifies potential strategies for their regulation in mammalian cells.

## Introduction

Heterotrimeric G-proteins function as molecular switches to regulate intracellular signaling pathways. The timing and duration of these signals are dependent on the lifetime of the activated (GTP-bound) Gα subunit. The conventional model for receptor-mediated G-protein activation is described herewith. In the basal state, a quiescent (GDP-bound) Gα subunit is complexed with a Gβγ heterodimer and coupled to the intracellular surface of a GPCR. Receptor activation by an extracellular stimulus results in the exchange of GTP for GDP on the Gα subunit and the dissociation of GTP-bound Gα from the Gβγ. This condition marks the activated (“ON”) state of receptor signaling, during which time the Gα and Gβγ subunits are free to engage downstream effector molecules, such as adenylyl cyclases, phospholipases, and ion channels. Effector signaling is terminated following Gα-catalyzed hydrolysis of GTP and reformation of the quiescent (“OFF”) state of the GPCR complex. Based on their membrane-spanning nature and their ability to transmit a wide array of extracellular signals to appropriate intracellular effector pathways, GPCRs represent one of the most important classes of therapeutic targets for drug discovery (Hopkins and Groom, 2002).

A less-well characterized pathway for the activation of G-protein α subunits involves regulatory proteins that reside on intracellular membranes. Some Gα subunits, such as Gαi3, for example, are localized primarily to intracellular membrane pools like the trans-Golgi network (TGN) (De Vries et al., 1998), LC3-positive autophagosomes (Garcia-Marcos et al., 2011), and the ERGIC, a trafficking compartment between the ER and the Cis-Golgi (Lo et al., 2015). A number of intracellular proteins can regulate the activity of Gαi3 at sites distal to the plasma membrane receptor complexes discussed above. G-protein dissociation inhibitors (GDIs) maintain the inactive signaling conformation of the Gαi/o subunits by stabilizing the GDP-bound state (Siderovski and Willard, 2005). One of the best known GDIs is AGS3, a protein found primarily on intracellular membranes at the Golgi, aggresomes, and LC3-positive autophagosomes (Vural et al., 2010; Garcia-Marcos et al., 2011; Oner et al., 2013). AGS3 can also prime Gαi/o subunits for activation by non-receptor guanine nucleotide exchange factors (GEFs), such as GIV (Lo et al., 2015). Importantly, GIV was shown to activate Gαi3 as a consequence of growth factor stimulation, supporting the premise that Gαi3 can be activated in a receptor-independent manner. Notably, GIV was found to be localized to the same compartments as Gαi3 including the plasma membrane, ERGIC, TGN, and AGS3-labeled autophagosomes (Garcia-Marcos et al., 2011; Lo et al., 2015). Another family of Gα subunit regulators, the RGS protein superfamily, functions as GTPase-activating proteins (GAPs) to inhibit G-protein signaling (Berman et al., 1996). There are more than 35 proteins in the human genome. All RGS proteins are typified by their ~120 aa GAP domain that is capable of increasing the rate of GTP hydrolysis by up to 2000-fold. They can be further classified into subgroups based on the organization of multiple modular protein–protein interaction domains that direct their localization to specific signaling complexes within the cell (Hollinger and Hepler, 2002). Like Gαi3, AGS3, and GIV, some RGS proteins are concentrated within intracellular membrane domains. Specifically, RGS19 is localized mainly to the TGN and plasma membrane where it can regulate Gαi3-mediated control of protein sorting and autophagic flux. We have previously shown that RGS4, a member of the regulators of G-protein signaling (RGS) protein superfamily, can traffic between plasma membrane, endosomes, Golgi/TGN membrane compartments within mammalian cells (Bastin et al., 2012). The ability of RGS4 to target different intracellular subdomains was highly dependent on the palmitoylation status of its amino-terminus. We showed that differential palmitoylation Cysteine2 and Cysteine12 had markedly different effects on its localization and function. Specifically, palmitoylation on Cys12 is critical for localization to the plasma membrane and inhibition of receptor-mediated G-protein signaling, whereas palmitoylation on Cys2 appears critical for allowing RGS4 to traffic into the intracellular endosome/Golgi pool. Together, these data suggested that RGS4 palmitoylation site mutants might be useful genetic tools for understanding the extent of plasma membrane versus intracellular G-protein activity that contributes to complex signaling, such as that by members of the MAPK family.

JNKs belong to the MAPK family. JNK isoforms have been implicated in the pathophysiology of a range of diseases, including Alzheimers disease (Akhter et al., 2015), arthritis (Schepetkin et al., 2015), obesity (Nakamura et al., 2015), diabetes (Dong et al., 2015), atherosclerosis (Kampschulte et al., 2014), abdominal aortic aneurysm (Zhang et al., 2015), cardiac disease (Sun et al., 2015), liver disease (Win et al., 2015), and tumorigenesis (Enomoto et al., 2015), making them an important target for therapeutic intervention. JNKs form part of a wider protein kinase cascade that mediates cellular responses to an array of stress-related stimuli including: heat shock (Ruan et al., 2015), hyperosmolarity (Gerke et al., 2014), ultraviolet irradiation (von Koschembahr et al., 2015), cytokine activity (Choi et al., 2015), and GPCRs (Yamauchi et al., 2000). Extracellular stress stimuli activate a cascade of kinases that involve mitogen-activated protein/ERK kinase kinase (MEKKs), mitogen-activated protein kinase kinase 4/7 (MKK4/7), and JNK1/2/3 assembled on intracellular scaffold proteins, such as JNK-interacting protein (JIP) (Whitmarsh, 2006). Once activated, JNKs can phosphorylate a large number of cellular proteins that are connected to stress signaling (Arthur and Ley, 2013). As their name suggests, JNKs are thought to be one of the most important activators of the transcription factor c-Jun via their ability to phosphorylate serines 63 and 73 within its transactivation domain (Li et al., 2004). JNKs may also activate other transcription factors, such as: JunD (Yazgan and Pfarr, 2002) and ATF2 (two members of the AP-1 transcription complex) (Gupta et al., 1995); hormone receptors (Caelles et al., 1997); FOXO4 (Essers et al., 2004); and PPARγ1 (Yin et al., 2006). Of importance to this work, JNKs have also been shown to phosphorylate a large number of protein substrates outside of the nucleus suggesting that they have important functions that are distinct from transcriptional regulation. Specifically, JNKs have been shown to regulate the following proteins in various compartments: cytoskeletal proteins – such as the microtubule-associated protein, tau (Yoshida et al., 2004); cytosolic proteins – including the E3 ligase, Itch (Gallagher et al., 2006), insulin-receptor substrate-1 (Hilder et al., 2003) and various 14-3-3 adaptors (Sunayama et al., 2005); mitochondrial proteins – such as Bcl2 (Yamamoto et al., 1999), Bad (Yu et al., 2004), and Bim (Lei and Davis, 2003); and plasma membrane-proximal components of focal adhesions – such as paxillin (Huang et al., 2003). It has been suggested that in order to phosphorylate such a wide variety of intracellular substrates, JNKs must be localized to the specific subcellular domains containing their substrates. Indeed, distinct pools of activated JNK have been identified in various intracellular compartments, including the nucleus (Kurinna et al., 2004), endosome/lysosome (Parameswaran et al., 2013), and mitochondrial fractions (Almeida et al., 2000); however, little is known about the neighborhood-specific molecules and pathways that are involved in regulating these unique signaling domains. We show here that the heterotrimeric G-protein, Gαi3, and the intracellular proteins that regulate its activity, such as AGS3 and RGS4, represent a novel signaling compartment that controls the local activity levels of JNK. These data provide strong rationale for characterization of the relative contribution of different intracellular JNK pools to the overall JNK activity profile of a cell or tissue, particularly when evaluating the significance of changing phosho-JNK (p-JNK) levels on Western blots from whole cell lysates.

## Materials and Methods

### Materials

The RGS4-YFP, RGS4-YFP (Cys2A and Cys12A), and RGS4-YFP (EN-AA) mutants were described previously by our group (Bastin et al., 2012; Bastin and Heximer, 2013). The EN-AA mutation in the GAP domain of RGS4 (E87A, N88) renders the protein catalytically inactive by preventing its interaction with Gαi (Srinivasa et al., 1998). HEK293 cells (tsA-201 derivative) were a kind gift from Zhong-Ping Feng (University of Toronto). All tissue culture media and transfection reagents were purchased from Invitrogen and Roche Scientific, respectively. Fluorescent-tagged versions of the TGN marker protein TGN38 were from J. Lippincott-Schwartz (National Institutes of Health, Bethesda, MD, USA). Gαi3-CFP construct was created by insertion of CFP within an intracellular loop of Gαi3 and was a kind gift from Catherine Berlot (Weis Center for Research, Geisinger Clinic, Danville, PA, USA). AGS3-GFP was a kind gift from Stephen Lanier (University of South Carolina). Antibodies against JNK and phospho-JNK were purchased from Cell Signaling with the respective catalog numbers: #9252S, #9251S. Horseradish peroxidase-coupled anti-rabbit secondary antibodies were from Cell Signaling (CAT # 70745) respectively. Unless otherwise stated, all other reagents and chemicals were from Sigma.

### Cell culture

HEK293 or MEF cells were grown in Dulbecco’s modified Eagle’s medium (DMEM):Ham’s F12 medium (1:1) (Gibco, respectively CAT # 11995-065 and # 11765-054), supplemented with 10% (v/v) heat-inactivated fetal bovine serum (Gibco, CAT # 12483020), 2 mM glutamine (Life Technology, CAT # 25030081), 10 μg/ml streptomycin, and 100 units/ml penicillin (Life Technology, CAT # 15140122) at 37°C in a humidified atmosphere with 5% CO_2_. To reduce the impact of growth factors and hormones on global cell signaling, cells were maintained in serum-free Earle’s Balanced Sodium Solution (EBSS, Life Technology, CAT #14155-063) supplemented with 200 mg/L of both CaCl_2_ and MgCl_2._

### Molecular biology

For subcellular localization studies, RGS4-YFP and cysteine point mutations expression plasmids were generated in the pEYFP-C1 as described previously (Bastin et al., 2012). Constitutively active Gαi3-R178C-CFP was created by site-directed mutagenesis methods using the forward strand primers 5′-cca act cag cca gat gtt ctt cgg aca tgt-3′ together with its reverse complement. The R178C mutation has been previously shown to impair the GTP hydrolysis activity by the α subunit, rendering the protein locked in its activated state. RGS proteins are however, capable of increasing the GTPase rate of these mutants. All plasmid constructs were purified using the Endofree Maxi kit (Qiagen, CAT # 12362) and verified by sequencing of the complete protein-coding region.

### Confocal microscopy

HEK293 cells were plated at 50% confluence in tissue culture-treated microscopy dishes (Ibidi, CAT # 81156) and transfected overnight with 1 μg of each construct to be tested using 2.5 μL of Xtremegene HP transfection reagent according to the manufacturer’s instructions (Roche, CAT # 06366236001). After 24 h, dishes were examined by confocal microscopy to determine their localization containing transfected cells. Spinning disk confocal microscopy was performed on live cells at 37°C in an environmental chamber maintained at 5% CO_2_ using a WaveFX Spinning-Disk Confocal Microscope (Quorum Technologies, Guelph, Canada), comprised of an Olympus IX81 microscope stand, a Yokogawa CSU10 spinning-disk unit, and a Hamamatsu C9100-13 EM-CCD camera, controlled by Volocity software. Imaging was performed using a 60×/1.42 N.A. oil immersion objective, using 405, 488, and 561 nm solid-state lasers for the excitation of CFP, YFP, and mRFP respectively. Z-stack intervals were 0.3–0.35 μm. Emission wavelength parameters of each were matched to the appropriate bandpass emission filters, and where more than one fluorescent channel was examined in a single cell, the possibility of bleed through fluorescence was excluded prior to evaluation of the co-localization of different proteins. All confocal images were collected and analyzed with the Volocity software package and figures were subsequently generated using Microsoft Office.

### Western blotting

Proteins were transferred to (Trans-Blot, BioRad) nitrocellulose membrane. Membranes were blocked for 1 h with Tris-buffered saline 0.1% Tween-20 (TBST) with 5% bovine serum albumin. Primary antibodies were diluted in TBST containing 5% BSA as per the vendor’s instructions and incubated with membranes overnight at 4°C before removing by washing. Horseradish peroxidase linked-secondary antibodies were diluted (1:3000) in TBST with 5% BSA was added for 2 h, before washing and signal detection using Super Signal West Pico Chemi-luminescent Substrate (Thermo Scientific). Western blots were analyzed by densitometry using Image J software analysis.

### Data collection, management, and statistical analysis

At the outset of each series of microscopy experiments, the experimenter was blinded to the identity of the transfectants until data collection and analysis were completed. Where indicated, one-way and two-way ANOVA with Tukey’s *post hoc* analysis were used to analyze the experimental results. *p < 0.05 was considered significant. Error bars depict standard error of the mean (SEM) for all graphs.

## Results and Discussion

**Figure 1:**
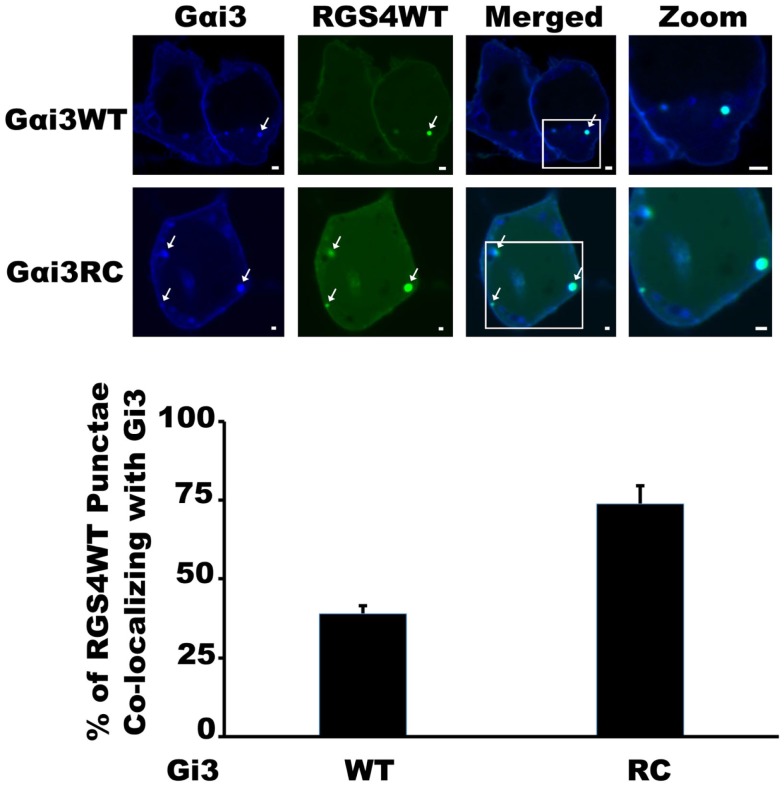
**RGS4 Shows preferential co-localization with Gαi3RC**. HEK293 cells were co-transfected with RGS4-YFP (yellow), and either Gαi3 (WT)- or Gαi3 (RC)-CFP (blue) to assess the extent of co-localization using spinning disk confocal microscopy. The merged view is a composite two-channel view of cells expressing the two indicated constructs. Data are representative of at least 100 dual-transfected cells. Arrows indicate co-localization on intracellular endosomal structures between RGS4 and Gαi3. Scale bars represent 1 μm.

**Figure 2:**
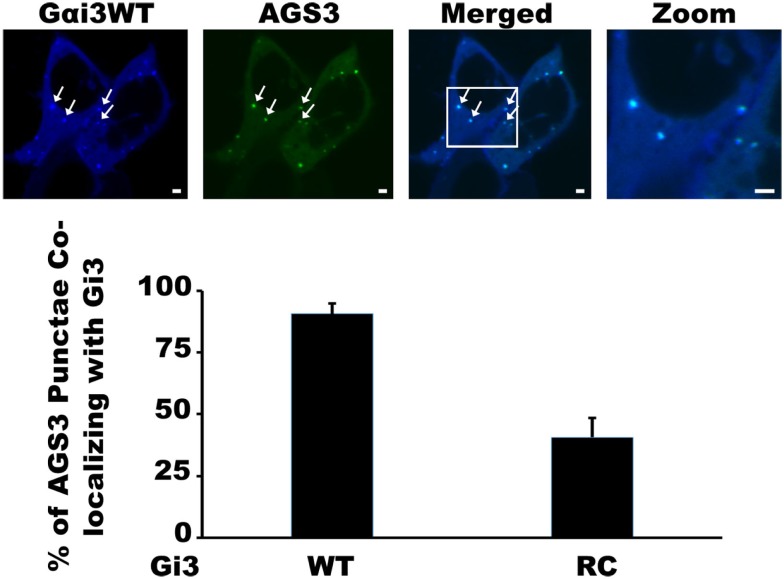
**Co-localization of Gαi3 with AGS3 in mammalian cells**. HEK 293 cells were co-transfected AGS3-GFP (green) and Gαi3WT-CFP (blue) to assess the extent of co-localization using spinning disk confocal microscopy. The merged view is a composite two-channel view of cells expressing the two indicated constructs. Data are representative of at least 100 dual-transfected cells. Arrows indicate co-localization between AGS3 and Gαi3 on intracellular endosomal structures. Scale bars represent 1 μm.

**Figure 3:**
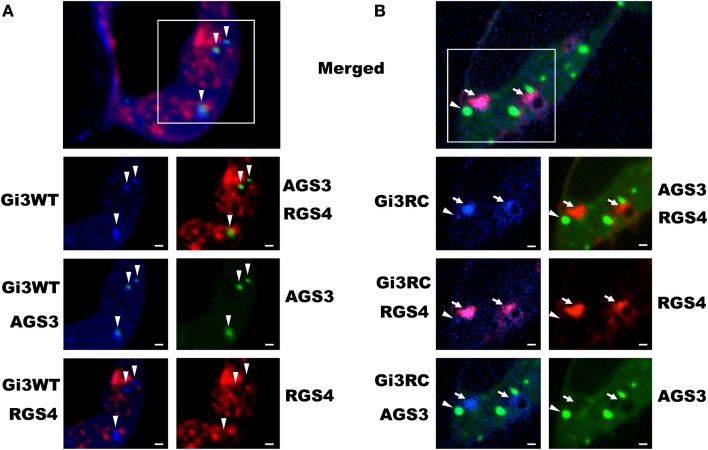
**Co-localization of Gαi3 with AGS3 and RGS4 is dependent on its activation state**. HEK 293 cells were co-transfected with RGS4-RFP (red), AGS3-GFP (green), and either Gαi3WT-CFP **(A)** or Gαi3RC-CFP **(B)** (blue) to assess the extent of co-localization using spinning disk confocal microscopy. The merged view is a composite three-channel view of cells expressing all three constructs. Shown below are the indicated single channel views or double-channel combinations. Data are representative of at least 100 triple-transfected cells. Arrows indicate co-localization between RGS4 and Gαi3, whereas arrowheads indicate co-localization between AGS3 and Gαi3. Scale bars represent 1 μm.

Our previous work with the RGS4 protein showed that there exists at least two distinct membrane-bound pools of RGS4 within mammalian cells (Bastin et al., 2012). These pools consisted of the known pool at the plasma membrane and a newly appreciated pool that targeted intracellular membranes, such as endosomes, Golgi, and other punctate structures (Bastin and Heximer, 2013). The demonstration that differential palmitoylation of cysteine residues in the amino-terminus of RGS4 could alter its distribution between these two membrane pools guided efforts to demonstrate potential functional differences between RGS4 at these different locations in the cell. While it was relatively straightforward to show that prevention of RGS4 trafficking to the plasma membrane via mutation of Cys12, the palmitoylation site adjacent to its membrane-targeting amphipathic helix, could inhibit the ability of RGS4 to inhibit Gq-mediated signaling from the plasma membrane, it was more complicated to demonstrate a functional consequence of the Cys2 mutation that prevented RGS4 localization to the intracellular membrane pool. Our attention turned to regulation of intracellular Gαi3 after we discovered overlapping expression of RGS4-YFP-containing punctae with those targeted by CFP-tagged Gαi3 (Figure 1). Notably, the extent of co-localization on intracellular punctae was typically greater (larger number of punctae/cell) between RGS4 and the constitutively active Gαi3 (R178C; RC) compared to Gαi3 (WT). A significant plasma membrane signal was also present for Gαi3, suggesting that like RGS4, Gαi3 may also traffic between the plasma membrane and the intracellular membrane compartments. These data suggested that intracellular RGS4 and Gαi3 may target some of the same intracellular domains to co-ordinately regulate specific intracellular signaling pathways. Consistent with previous reports, Gαi3 and AGS3 were also found together on intracellular membrane structures in our expression system (Figure 2). Notably, in the presence of AGS3, we observed dramatically reduced co-localization of RGS4 and WT Gαi3 compared to Gαi3 (RC) (marked by arrowheads in Figures 3A,B). Together, these data suggested that Gαi3 may shuttle between AGS3-containing (GDP-bound Gαi3) and RGS4-containing (GTP-bound Gαi3) compartments depending on its state of activation. These data simply reflect the preferences of the RGS box for GTP-bound Gαi3 and AGS3 GPR/GoLoco motifs for inactive/GDP-bound Gαi3; however, preliminary evidence to argue against this notion comes from experiments showing the catalytically dead RGS4 (EN-AA) mutant had similar co-localization with Gαi3 (RC)-containing punctae as wild-type RGS4. Moreover, the co-expression of Gαi3 with either RGS4 or AGS3 did not alter their localization in any discernable manner. These data suggest that these proteins traffic together on similar endosome-like structures where they may be co-localized, without necessarily interacting stably with one another. Such a system would allow RGS4 to fine tune the levels of Gαi3 activity, while they are in the same compartment and then pass off inactive Gαi3-GDP to another membrane compartment (presumably an AGS3-containing one), where Gαi3 could be primed for reactivation.

**Figure 4:**
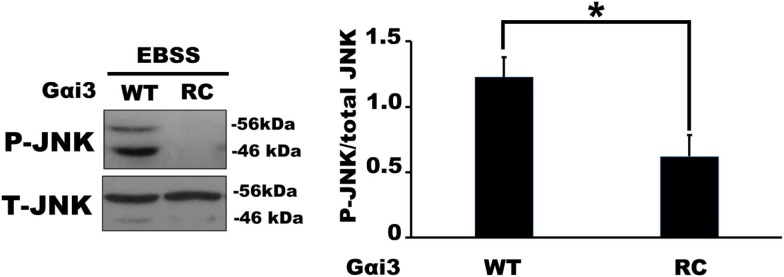
**Activated Gαi3 reduces the levels of phospho(p)-JNK in HEK 293 cells**. Relative changes in endogenous p-JNK/total JNK ratio were quantified in the presence of Gαi3-CFP (WT) and (RC) by Western Blotting. The left panel show a representative Western blot of p-JNK/total JNK regulation by Gαi3 (WT) and (RC) expression. The right panel shows the quantification of p-JNK/total JNK ratio in four independent experiments (one-way ANOVA with Tukey *post hoc* test *P < 0.01).

**Figure 5:**
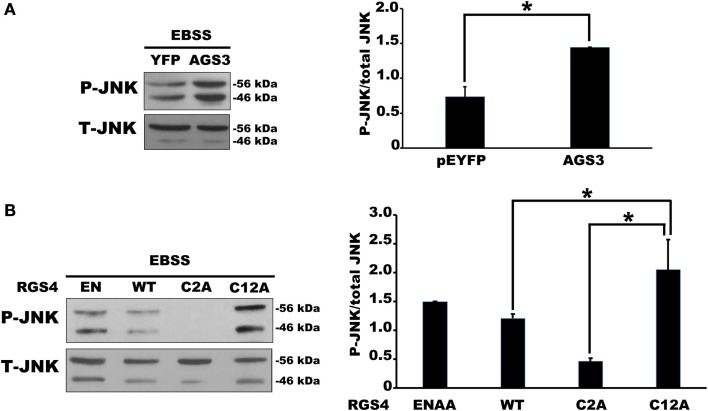
**Expression of AGS3 and intracellular RGS4 increased phospho(p)-JNK in HEK 293 cells**. **(A)** Relative changes in endogenous p-JNK/total JNK ratio were quantified in the presence of AGS3 or empty vector as indicated. The left panel shows a representative Western blot of p-JNK/total JNK regulation by AGS3 expression. The right panel shows the quantification of p-JNK/total JNK ratio in four independent experiments. **(B)** The effect of RGS activity of RGS4 on p-JNK/total JNK was observed by Western blot with the catalytic dead mutant RGS4 (ENAA). The effect of palmitoylation site of RGS4 on the variation of p-JNK/total JNK was observed by Western blot with the expression of Cys2 and Cys12 mutants. The left panel shows a representative Western blot of p-JNK/total JNK regulation by the indicated constructs. The right panel shows the quantification of p-JNK/total JNK ratio in four independent experiments [one-way ANOVA with *post hoc* Tukey *P < 0.005 for **(A)**, and *P < 0.05 for **(B)**].

**Figure 6:**
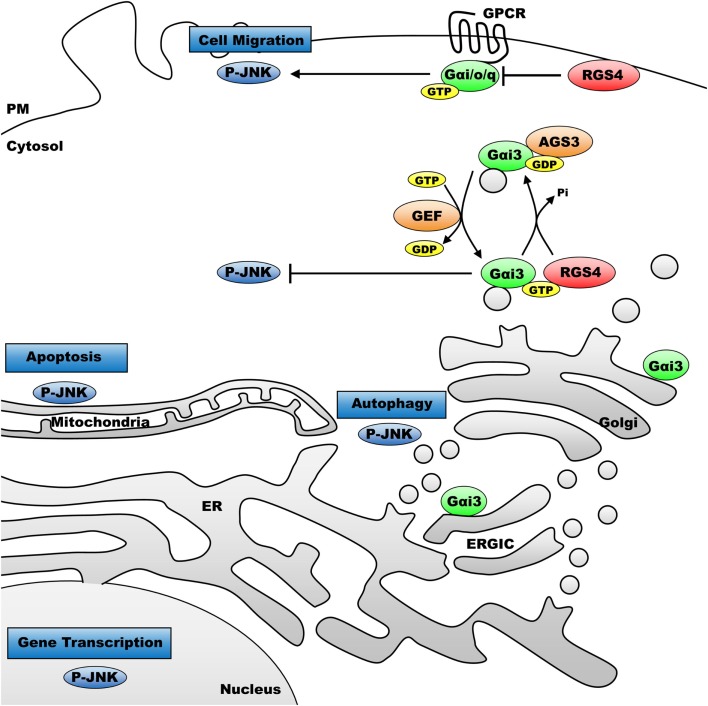
**Model for intracellular functions of p-JNK and the effect of heterotrimeric G-proteins on p-JNK accordingly to the cellular compartment**. P-JNK has been correlated to distinct functions accordingly to its intracellular compartments. Indeed, p-JNK regulates gene transcription upon nuclear localization, apoptosis when it targets mitochondria. P-JNK also regulates signals located at the ER–Golgi vicinity to enhance autophagy and at the plasma membrane to regulate cell migration. Heterotrimeric G-protein coupled receptor activation at the plasma membrane is known to increase levels of p-JNK in mammalian cells. Here, we showed that activated Gαi3 protein significantly inhibits p-JNK and inhibitors of Gαi/o proteins, AGS3 and RGS4, promote p-JNK when located on intracellular compartments. Conversely, RGS4 located selectively at the plasma membrane potently inhibited p-JNK.

We next examined JNK activity, an intracellular signaling pathway that we expected to be sensitive to changes in Gαi3 activity. Indeed, the prediction, based on previous reports where JNK was activated downstream of various GPCRs, was that increased Gαi3 activity would increase the level of intracellular JNK activation as determined by Western blotting for phosphorylated JNK (p-JNK). Surprisingly, however, the relative activity level of Gαi3 in our system inversely correlated with the observed p-JNK levels. Specifically, Gαi3 (RC) mediated a profound reduction of p-JNK in our system compared to Gαi3 (WT) (Figure 4). As expected, both Gαi3 clones showed a reduced level of p-JNK relative to empty vector controls (data not shown). Together, these data suggested the novel premise that Gαi3 signaling inhibits intracellular JNK activation. Since, AGS3 functions as a GDI on intracellular membrane pools, we next examined whether AGS3-mediated stabilization of GDP-bound (inactive) Gαi3 may also regulate JNK activation. Consistent with our new model, AGS3 expression markedly increased the levels of p-JNK observed in our cells relative to YFP control (Figure 5). These data suggest that there exists a tonic level of endogenous Gαi-mediated JNK inhibition in HEK293 cells that can be modulated by the expression of AGS3 or other similar GDI partners. In support of this, we also found that pertussis toxin increased p-JNK levels in a dose-dependent fashion (data not shown). Finally, we examined the effect of another potent Gαi3 inhibitor, RGS4, on the regulation of JNK activity in our system (Figure 5). Endogeneous Gαi3 with wild-type RGS4 expression resulted in a modest decrease of p-JNK compared to expression of its catalytically inactive EN-AA mutant. At first glance, these data seemed inconsistent with the observations for AGS3 and Gαi3 above. However, a more compelling story emerged when we examined the effects of the RGS4 palmitoylation site mutants Cys2A and Cys12A on intracellular JNK signaling. Notably, for the Cys2 mutant, when RGS4 was nearly exclusively localized to the plasma membrane (i.e., unable to target the intracellular membrane pool), there was a marked decrease in p-JNK levels. By contrast, for the Cys12A mutant when RGS4 was nearly exclusively localized to intracellular membranes, there was a marked increase in p-JNK levels to those even exceeding the levels observed for the catalytically inactive (EN-AA) RGS4 construct. Taken together, these data suggest that total JNK signaling in a cell or tissue represents a combination of JNK pools that likely includes cytosolic, nuclear, plasma membrane, and intracellular fractions. Wild-type RGS4, by virtue of its ability to target and inhibit multiple intracellular signaling pools showed a much different effect on JNK signaling compared to either of the two individual palmitoylation site mutants. It should be noted, however, that we cannot rule out the possibility that mutation of Cys2 and its effects on RGS4 stability, via preventing N-end rule degradation of the protein may also have contributed to its ability to regulate intracellular JNK activity. A mechanistic model showing the spatial distribution of JNK, Gαi3, and its regulators is presented in Figure 6.

It is important to note that the mechanism by which Gαi3 inhibits intracellular JNK remains to be identified. It will be critical to determine whether this unique effect is mediated by direct protein–protein interaction between Gαi3 and JNK, or alternatively whether other effector pathways downstream of activated Gαi3, such as adenylyl cyclases or PI3Ks, may be involved. Although the effects of Gαi3 (RC) are consistent with an effect mediated by the α subunit, further studies will be required to determine whether βγ or other βγ-like partners may also participate in the complexes that regulate intracellular JNK activity. Moreover, while it appears likely that the effects of AGS3 and pertussis toxin are exerted via their activity on Gαi3, we cannot rule out the possibility that other endogenous Gαi subunits may also contribute to intracellular JNK regulation. These new findings may also be useful in the design of strategies to identify and selectively target specific intracellular JNK pools and their associated physiologic activities. At this stage, it remains to be determined whether the JNK pool regulated by Gαi3 in our system is associated with one of the known regulatory functions of JNK, such as apoptosis, cell migration, transcription, autophagy, and ER–Golgi trafficking, or whether it has a yet undiscovered function. It seems likely, however, based on the known localization and intracellular function of Gαi3 that this pool of JNK will be somehow linked to intracellular membrane trafficking at the level of the ER–Golgi and their associated vesicular compartments.

It may be important to determine whether this signaling domain contains one or more of the different known JNK isoforms. Mammalian JNKs are encoded by three distinct genes (Jnk1, Jnk2, and Jnk3). Alternative splicing generates up to 10 different protein products varying in size from 46 to 55 kDa, all of which have been sequenced and analyzed for possible specificity determinants. Despite their the high level of homology (>80%), differences between amino- and carboxy-terminal sequence or exon usage suggest the existence of functional specificity (Gupta et al., 1996; Guo and Whitmarsh, 2008). While isoform-specific knockouts and the development of pan-specific JNK inhibitors have thus far been very useful in the study of JNK function (Gehringer et al., 2015), the simple fact remains that for most JNK substrates, there is still very little information regarding isoform-specific affinity. As suggested by our data above, targeting discreet intracellular protein complexes, such as endosomal Gαi3, or its regulators and effectors, may offer unique molecular strategies for modulating JNK activity. One such strategy might be to alter the plasma membrane versus endosomal distribution profile of the Gαi3 regulator RGS4. This might be accomplished by promoting site-selective palmitoylation of its amino-terminus. Currently, studies are underway to evaluate the specificity of the various palmitoyl-CoA transferases (DHHC family proteins) for Cys2 and Cys12 with this in mind. It is also interesting that the amino-terminal domain of Gαi3 itself requires palmitoylation for its optimal membrane targeting and function. Thus, regulation of Gαi3 by specific DHHC isoforms might provide another useful access point for regulating the intracellular JNK pool. Lastly, modulation of AGS3 activity may provide another unique opportunity to modulate intracellular JNK via Gαi3. Using structural techniques and data, other groups have identified the residues within AGS3 that allow its binding (Peterson et al., 2002; Willard et al., 2008) and GDI activity (Kimple et al., 2002, 2004) for Gαi. Intriguingly, these data may ultimately inform the design of peptide mimetics that selectively could interfere with AGS3–Gαi3 interactions to regulate Gαi3 activity and local JNK regulation.

In summary, we have uncovered a new role for Gαi3-mediated function in mammalian cells – specifically as an inhibitor of JNK activation and signaling. The model for the proposed mechanism is shown in Figure 6. Activation of Gαi3 (in this case via receptor-independent GEF activity) on intracellular membrane pools leads to inhibition of local JNK activation. Then, illustrated, the regulation Gαi3 activity (cycling between different nucleotide states) may be coordinated by other intracellular players, including AGS3, RGS proteins, and GIV/girdin (GEF). Changes in intracellular JNK activity may then alter physiologic activities at one or more of the other intracellular locations where unique JNK pools are thought to be important, such as mitochondria (apoptosis), ER–Golgi interface (autophagy), or the nucleus (transcriptional programs). This work supports further characterization of the novel protein complexes regulated by heterotrimeric G-proteins that are found localized to intracellular membrane pools deep within the cell, distal to the influences of most surface GPCRs. The characterization of receptor-independent G-protein activation and signaling, and its relationship to cellular health and homeostasis is an important emerging area of investigation that is likely to reveal a number of new cellular functions for Gα subunits, as well as their intracellular binding partners and effectors.

## Conflict of Interest Statement

The authors declare that the research was conducted in the absence of any commercial or financial relationships that could be construed as a potential conflict of interest.

## Abbreviations

AGS, activator of G-protein signaling; AP-1, activator protein 1; ATF2, activating transcription factor 2; BAD, Bcl2-associated death promoter; Bcl2, B-cell lymphoma 2; BIM, Bcl2 interacting mediator of cell death; CFP, cyan fluorescent protein; DHHC, Asp-His-His-Cys containing motif palmitoylating enzyme; DHHC, palmitoyl-CoA transferase active site motif; EBSS, Earle’s balanced sodium solution; ER, endoplasmic reticulum; ERGIC, ER–Golgi intermediate compartment; FOXO4, forkhead box protein O4; GAIP, G alpha interacting protein; GAP, GTPase-activating protein; GDI, guanosine nucleotide dissociation inhibitor; GDP, guanosine-diphosphate; GEF, guanine nucleotide dissociation inhibitor; GFP, green fluorescent protein; GPCR, G-protein coupled receptor; G-protein, guanine nucleotide binding protein; GTP, guanosine-5,-triphosphate; HEK, human embryonic kidney; JNK, c-Jun N-terminal kinase; MAPK, mitogen-activated protein kinase; MEKK, mitogen-activated protein/ERK kinase kinases; MKK, mitogen-activated protein kinase kinase; MKK4/7, mitogen-activated protein kinase kinase 4/7; PI3K, phophoinositide 3-kinase; PM, plasma membrane; RFP, red fluorescent protein; RGS, regulator of G-protein signaling; TGN, trans-Golgi network; WT, wild type; YFP, yellow fluorescent protein.

## References

[B1] Akhter R. Sanphui P. Das H. Saha P. Biswas S. C. (2015). The regulation of p53 up-regulated modulator of apoptosis by JNK/c-Jun pathway in beta Amyloid-induced neuron death. J. Neurochem. 10.1111/jnc.13128 25891762

[B2] Almeida E. A. Ilic D. Han Q. Hauck C. R. Jin F. Kawakatsu H. (2000). Matrix survival signalling: from fibronectin via focal adhesion kinase to c-Jun NH(2)-terminal kinase. J. Cell Biol. 149, 741–754. 10.1083/jcb.149.3.741 10791986 PMC2174844

[B3] Arthur J. S. Ley S. C. (2013). Mitogen-activated protein kinases in innate immunity. Nat. Rev. Immunol. 13, 679–692. 10.1038/nri3495 23954936

[B4] Bastin G. Heximer S. (2013). Rab family protein regulate the endosomal trafficking and function of RGS4. J. Biol. Chem. 288, 21836–21849. 10.1074/jbc.M113.466888 23733193 PMC3724640

[B5] Bastin G. Singh K. Dissanayake K. Mighiu A. S. Nurmohamed A. Heximer S. P. (2012). Amino-terminal cysteine residues differentially influence RGS4 protein plasma membrane targeting, intracellular trafficking, and function. J. Biol. Chem. 287, 28966–28974. 10.1074/jbc.M112.345629 22753418 PMC3436580

[B6] Berman D. M. Wilkie T. M. Gilman A. G. (1996). GAIP and RGS4 are GTPase-activating proteins for the Gi subfamily of G protein alpha subunits. Cell 86, 445–452. 10.1016/S0092-8674(00)80117-8 8756726

[B7] Caelles C. Gonzalez-Sancho J. M. Munoz A. (1997). Nuclear hormone receptor antagonism with AP-1 by inhibition of the JNK pathway. Genes Dev. 11, 3351–3364. 10.1101/gad.11.24.3351 9407028 PMC316827

[B8] Choi H. Dikalova A. Stark R. J. Lamb F. S. (2015). c-Jun N-terminal kinase attenuates TNFalpha signalling by reducing Nox1-dependent endosomal ROS production in vascular smooth muscle cells. Free Radic. Biol. Med. 86, 219–227. 10.1016/j.freeradbiomed.2015.05.015 26001727

[B9] De Vries L. Elenko E. McCaffery J. M. Fischer T. Hubler L. McQuistan T. (1998). RGS-GAIP, a GTPase-activating protein for Galphai heterotrimeric G proteins, is located on clathrin-coated vesicles. Mol. Biol. Cell 9, 1123–1134. 10.1091/mbc.9.3.599 9571244 PMC25334

[B10] Dong Y. Gao G. Fan H. Li S. Li X. Liu W. (2015). Activation of the liver X receptor by agonist TO901317 improves hepatic insulin resistance via suppressing reactive oxygen species and JNK pathway. PLoS ONE 10:e0124778. 10.1371/journal.pone.0124778 25909991 PMC4409387

[B11] Enomoto M. Kizawa D. Ohsawa S. Igaki T. (2015). JNK signalling is converted from anti- to pro-tumor pathway by Ras-mediated switch of Warts activity. Dev. Biol. 403, 162–171. 10.1016/j.ydbio.2015.05.001 25967126

[B12] Essers M. A. Weijzen S. de Vries-Smits A. M. Saarloos I. de Ruiter N. D. Bos J. L. (2004). FOXO transcription factor activation by oxidative stress mediated by the small GTPase Ral and JNK. EMBO J. 23, 4802–4812. 10.1038/sj.emboj.7600476 15538382 PMC535088

[B13] Gallagher E. Gao M. Liu Y. C. Karin M. (2006). Activation of the E3 ubiquitin ligase itch through a phosphorylation-induced conformational change. Proc. Natl. Acad. Sci. U.S.A. 103, 1717–1722. 10.1073/pnas.0510664103 16446428 PMC1413664

[B14] Garcia-Marcos M. Ear J. Farquhar M. G. Ghosh P. (2011). A GDI (AGS3) and a GEF (GIV) regulate autophagy by balancing G protein activity and growth factor signals. Mol. Biol. Cell 22, 673–686. 10.1091/mbc.E10-08-0738 21209316 PMC3046063

[B15] Gehringer M. Muth F. Koch P. Laufer S. A. (2015). c-Jun N-terminal kinase inhibitors: a patent review (2010-2014). Expert Opin. Ther. Pat. 25, 849–872. 10.1517/13543776.2015.1039984 25991433

[B16] Gerke P. Keshet A. Mertenskotter A. Paul R. J. (2014). The JNK-like MAPK KGB-1 of *Caenorhabditis elegans* promotes reproduction, lifespan, and gene expressions for protein biosynthesis and germline homeostasis but interferes with hyperosmotic stress tolerance. Cell. Physiol. Biochem. 34, 1951–1973. 10.1159/000366392 25500773

[B17] Guo C. Whitmarsh A. J. (2008). The beta-arrestin-2 scaffold protein promotes c-Jun N-terminal kinase-3 activation by binding to its nonconserved N terminus. J. Biol. Chem. 283, 15903–15911. 10.1074/jbc.M710006200 18408005 PMC3259632

[B18] Gupta S. Barrett T. Whitmarsh A. J. Cavanagh J. Sluss H. K. Derijard B. (1996). Selective interaction of JNK protein kinase isoforms with transcription factors. EMBO J. 15, 2760–2770. 8654373 PMC450211

[B19] Gupta S. Campbell D. Derijard B. Davis R. J. (1995). Transcription factor ATF2 regulation by the JNK signal transduction pathway. Science 267, 389–393. 10.1126/science.7824938 7824938

[B20] Hilder T. L. Tou J. C. Grindeland R. E. Wade C. E. Graves L. M. (2003). Phosphorylation of insulin receptor substrate-1 serine 307 correlates with JNK activity in atrophic skeletal muscle. FEBS Lett. 553, 63–67. 10.1016/S0014-5793(03)00972-4 14550547

[B21] Hollinger S. Hepler J. R. (2002). Cellular regulation of RGS proteins: modulators and integrators of G protein signalling. Pharmacol. Rev. 54, 527–559. 10.1124/pr.54.3.527 12223533

[B22] Hopkins A. L. Groom C. R. (2002). The druggable genome. Nat. Rev. Drug Discov. 1, 727–730. 10.1038/nrd892 12209152

[B23] Huang C. Rajfur Z. Borchers C. Schaller M. D. Jacobson K. (2003). JNK phosphorylates paxillin and regulates cell migration. Nature 424, 219–223. 10.1038/nature01745 12853963

[B24] Kampschulte M. Stockl C. Langheinrich A. C. Althohn U. Bohle R. M. Krombach G. A. (2014). Western diet in ApoE-LDLR double-deficient mouse model of atherosclerosis leads to hepatic steatosis, fibrosis, and tumorigenesis. Lab. Invest. 94, 1273–1282. 10.1038/labinvest.2014.112 25199052

[B25] Kimple R. J. Kimple M. E. Betts L. Sondek J. Siderovski D. P. (2002). Structural determinants for GoLoco-induced inhibition of nucleotide release by Galpha subunits. Nature 416, 878–881. 10.1038/416878a 11976690

[B26] Kimple R. J. Willard F. S. Hains M. D. Jones M. B. Nweke G. K. Siderovski D. P. (2004). Guanine nucleotide dissociation inhibitor activity of the triple GoLoco motif protein G18: alanine-to-aspartate mutation restores function to an inactive second GoLoco motif. Biochem. J. 378(Pt 3), 801–808. 10.1042/bj20031686 14656218 PMC1224015

[B27] Kurinna S. M. Tsao C. C. Nica A. F. Jiffar T. Ruvolo P. P. (2004). Ceramide promotes apoptosis in lung cancer-derived A549 cells by a mechanism involving c-Jun NH2-terminal kinase. Cancer Res. 64, 7852–7856. 10.1158/0008-5472.CAN-04-1552 15520191

[B28] Lei K. Davis R. J. (2003). JNK phosphorylation of Bim-related members of the Bcl2 family induces Bax-dependent apoptosis. Proc. Natl. Acad. Sci. U.S.A. 100, 2432–2437. 10.1073/pnas.2332656100 12591950 PMC151358

[B29] Li L. Feng Z. Porter A. G. (2004). JNK-dependent phosphorylation of c-Jun on serine 63 mediates nitric oxide-induced apoptosis of neuroblastoma cells. J. Biol. Chem. 279, 4058–4065. 10.1074/jbc.M400525200 14617628

[B30] Lo I. C. Gupta V. Midde K. K. Taupin V. Lopez-Sanchez I. Kufareva I. (2015). Activation of Galphai at the Golgi by GIV/girdin imposes finiteness in Arf1 signalling. Dev. Cell 33, 189–203. 10.1016/j.devcel.2015.02.009 25865347 PMC4415880

[B31] Nakamura T. Kunz R. C. Zhang C. Kimura T. Yuan C. L. Baccaro B. (2015). A critical role for PKR complexes with TRBP in immunometabolic regulation and eIF2alpha phosphorylation in obesity. Cell Rep. 11, 295–307. 10.1016/j.celrep.2015.03.021 25843719 PMC4439210

[B32] Oner S. S. Vural A. Lanier S. M. (2013). Translocation of activator of G-protein signalling 3 to the Golgi apparatus in response to receptor activation and its effect on the trans-Golgi network. J. Biol. Chem. 288, 24091–24103. 10.1074/jbc.M112.444505 23770668 PMC3745352

[B33] Parameswaran N. Enyindah-Asonye G. Bagheri N. Shah N. B. Gupta N. (2013). Spatial coupling of JNK activation to the B cell antigen receptor by tyrosine-phosphorylated ezrin. J. Immunol. 190, 2017–2026. 10.4049/jimmunol.1201292 23338238 PMC3578034

[B34] Peterson Y. K. Hazard S. III Graber S. G. Lanier S. M. (2002). Identification of structural features in the G-protein regulatory motif required for regulation of heterotrimeric G-proteins. J. Biol. Chem. 277, 6767–6770. 10.1074/jbc.C100699200 11756403

[B35] Ruan J. Qi Z. Shen L. Jiang Y. Xu Y. Lan L. (2015). Crosstalk between JNK and NF-kappaB signalling pathways via HSP27 phosphorylation in HepG2 cells. Biochem. Biophys. Res. Commun. 456, 122–128. 10.1016/j.bbrc.2014.11.045 25446109

[B36] Schepetkin I. A. Kirpotina L. N. Hammaker D. Kochetkova I. Khlebnikov A. I. Lyakhov S. A. (2015). Anti-inflammatory effects and joint protection in collagen-induced arthritis after treatment with IQ-1S, a selective c-Jun N-terminal kinase inhibitor. J. Pharmacol. Exp. Ther. 353, 505–516. 10.1124/jpet.114.220251 25784649 PMC4429673

[B37] Siderovski D. P. Willard F. S. (2005). The GAPs, GEFs, and GDIs of heterotrimeric G-protein alpha subunits. Int. J. Biol. Sci. 1, 51–66. 10.7150/ijbs.1.51 15951850 PMC1142213

[B38] Srinivasa S. P. Watson N. Overton M. C. Blumer K. J. (1998). Mechanism of RGS4, a GTPase-activating protein for G protein alpha subunits. J. Biol. Chem. 273, 1529–1533. 10.1074/jbc.273.3.1529 9430692

[B39] Sun F. Duan W. Zhang Y. Zhang L. Qile M. Liu Z. (2015). Simvastatin alleviates cardiac fibrosis induced by infarction via up-regulation of transforming growth factor, beta receptor III expression. Br. J. Pharmacol. 172, 3779–3792. 10.1111/bph.13166 25884615 PMC4523335

[B40] Sunayama J. Tsuruta F. Masuyama N. Gotoh Y. (2005). JNK antagonizes Akt-mediated survival signals by phosphorylating 14-3-3. J. Cell Biol. 170, 295–304. 10.1083/jcb.200409117 16009721 PMC2171419

[B41] von Koschembahr A. M. Swope V. B. Starner R. J. Abdel-Malek Z. A. (2015). Endothelin-1 protects human melanocytes from UV-induced DNA damage by activating JNK and p38 signalling pathways. Exp. Dermatol. 24, 269–274. 10.1111/exd.12638 25607638

[B42] Vural A. Oner S. An N. Simon V. Ma D. Blumer J. B. (2010). Distribution of activator of G-protein signalling 3 within the aggresomal pathway: role of specific residues in the tetratricopeptide repeat domain and differential regulation by the AGS3 binding partners Gi(alpha) and mammalian inscuteable. Mol. Cell. Biol. 30, 1528–1540. 10.1128/MCB.01018-09 20065032 PMC2832490

[B43] Whitmarsh A. J. (2006). The JIP family of MAPK scaffold proteins. Biochem. Soc. Trans. 34(Pt 5), 828–832. 10.1042/BST0340828 17052208

[B44] Willard F. S. Zheng Z. Guo J. Digby G. J. Kimple A. J. Conley J. M. (2008). A point mutation to Galphai selectively blocks GoLoco motif binding: direct evidence for Galpha.GoLoco complexes in mitotic spindle dynamics. J. Biol. Chem. 283, 36698–36710. 10.1074/jbc.M804936200 18984596 PMC2605979

[B45] Win S. Than T. A. Le B. H. Garcia-Ruiz C. Fernandez-Checa J. C. Kaplowitz N. (2015). Sab (Sh3bp5) dependence of JNK mediated inhibition of mitochondrial respiration in palmitic acid induced hepatocyte lipotoxicity. J. Hepatol. 62, 1367–1374. 10.1016/j.jhep.2015.01.032 25666017 PMC4439305

[B46] Yamamoto K. Ichijo H. Korsmeyer S. J. (1999). BCL-2 is phosphorylated and inactivated by an ASK1/Jun N-terminal protein kinase pathway normally activated at G(2)/M. Mol. Cell. Biol. 19, 8469–8478. 10567572 10.1128/mcb.19.12.8469 PMC84954

[B47] Yamauchi J. Kawano T. Nagao M. Kaziro Y. Itoh H. (2000). Gi-dependent activation of c-Jun N-terminal kinase in human embryonal kidney 293 cells. J. Biol. Chem. 275, 7633–7640. 10.1074/jbc.275.11.7633 10713072

[B48] Yazgan O. Pfarr C. M. (2002). Regulation of two JunD isoforms by Jun N-terminal kinases. J. Biol. Chem. 277, 29710–29718. 10.1074/jbc.M204552200 12052834

[B49] Yin R. Dong Y. G. Li H. L. (2006). PPARgamma phosphorylation mediated by JNK MAPK: a potential role in macrophage-derived foam cell formation. Acta Pharmacol. Sin. 27, 1146–1152. 10.1111/j.1745-7254.2006.00359.x 16923334

[B50] Yoshida H. Hastie C. J. McLauchlan H. Cohen P. Goedert M. (2004). Phosphorylation of microtubule-associated protein tau by isoforms of c-Jun N-terminal kinase (JNK). J. Neurochem. 90, 352–358. 10.1111/j.1471-4159.2004.02479.x 15228592

[B51] Yu C. Minemoto Y. Zhang J. Liu J. Tang F. Bui T. N. (2004). JNK suppresses apoptosis via phosphorylation of the proapoptotic Bcl-2 family protein BAD. Mol. Cell 13, 329–340. 10.1016/S1097-2765(04)00083-8 14967141

[B52] Zhang X. J. He C. Tian K. Li P. Su H. Wan J. B. (2015). Ginsenoside Rb1 attenuates angiotensin II-induced abdominal aortic aneurysm through inactivation of the JNK and p38 signalling pathways. Vascul. Pharmacol. 10.1016/j.vph.2015.04.003 25912763

